# Acute Vaccine-Related Encephalopathy and Acute Disseminated Encephalomyelitis (ADEM) After COVID-19 Vaccination: A Case Series From Bangladesh

**DOI:** 10.7759/cureus.39724

**Published:** 2023-05-30

**Authors:** Muhammad Rezeul Huq, Ghulam Kawnayn, Humayun Kabir, Md. Ismail Chowdhury, Mahin Binte Anwar

**Affiliations:** 1 Neurology, Combined Military Hospital, Dhaka, BGD; 2 Radiology, Combined Military Hospital, Dhaka, BGD

**Keywords:** case series, case report, acute vaccine-related encephalopathy, acute demyelinating encephalomyelitis, adem, covid-19 vaccine, neurological complications

## Abstract

There are several reported cases of various neurological adverse effects following the COVID-19 vaccination globally. Acute vaccine-related encephalopathy and acute disseminated encephalomyelitis (ADEM) are included among them. Here we are reporting three cases of ADEM and one case of acute vaccine-related encephalopathy from Bangladesh, which have a possible association with COVID-19 vaccines. All three ADEM cases were elderly; two cases developed symptoms after receiving the second dose of the Sinopharm vaccine, and another case after receiving the second dose of the Sinovac vaccine. We have treated another case of acute vaccine-related encephalopathy following receiving the Moderna vaccine. The patients had features of encephalopathy, including altered consciousness and convulsions. The ADEM cases had MRI (magnetic resonance imaging) brain findings suggestive of ADEM. The other case had normal MRI findings. All the cases were treated with intravenous corticosteroids with full recovery, except for one ADEM patient, who developed aspiration pneumonia and died. Though it is not possible to conclude that COVID-19 vaccination is the causative agent behind these cases, this case series will help to increase awareness regarding the early detection and treatment of these serious adverse effects.

## Introduction

Neurological complications are well-known after vaccination [1]. Mass vaccination after the COVID-19 pandemic also led to various complications. Among these complications, encephalitis, encephalopathy, or acute disseminated encephalomyelitis (ADEM) may also occur [2,3,4]. Encephalopathy is a clinical syndrome due to numerous etiologies having clinical features of altered mental status affecting patients’ cognition or level of arousal [5]. Acute encephalopathy is a possible complication after the COVID-19 vaccination [2,3,4]. ADEM is an inflammatory demyelinating disorder of the central nervous system involving the brain, spinal cord, and optic nerve. Children are more affected than adults. The patients may have variable clinical features, including encephalopathy (altered behavior, decreased consciousness, or irritability), with variable neurological deficits. Sometimes fever, seizures, and features of meningism may be present, mimicking infectious meningoencephalitis. However, ADEM has very characteristic magnetic resonance imaging (MRI) features that help to differentiate it from other differentials like infective encephalitis, autoimmune encephalitis, and other demyelinating diseases [6].

In this case series, we report a total of four cases of ADEM and acute reversible encephalopathy possibly associated with Moderna, Sinopharm, and Sinovac vaccines. Though we had certain limitations regarding completing necessary investigations, the temporal profile and clinical scenario were suggestive of the possible role of COVID-19 vaccination in these cases. This study will add important information regarding COVID-19 vaccination-related neurological adverse effects.

## Case presentation

Case one

**Figure 1:**
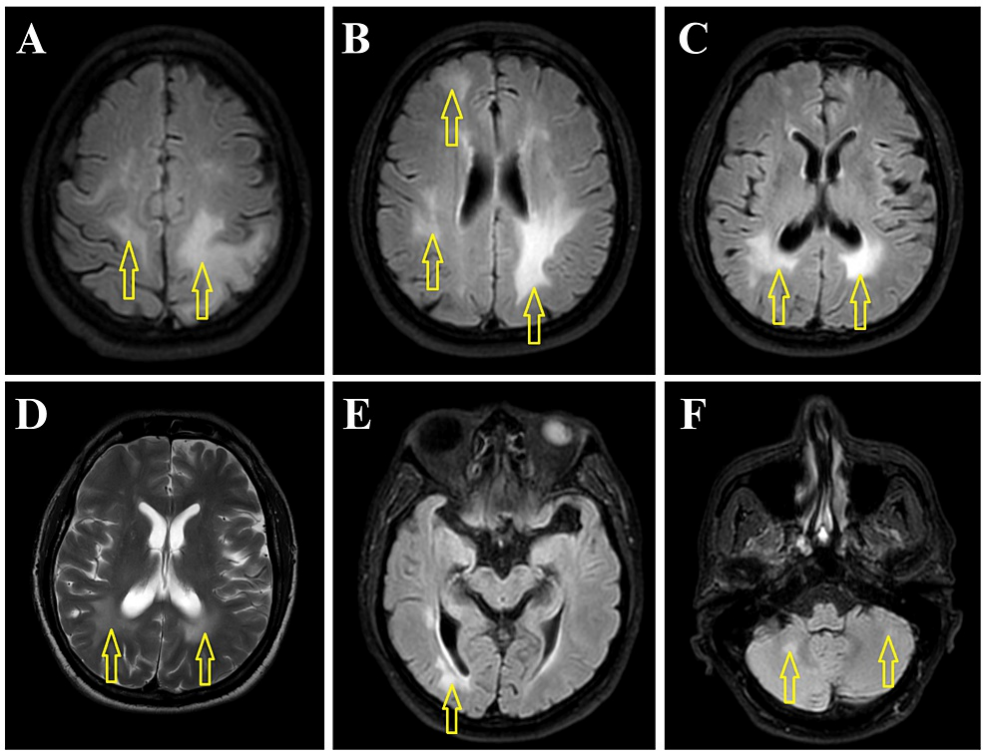
Axial FLAIR (1A, 1B, 1C, 1E, 1F) and T2W (1D) MRIs of the brain at different levels show bilateral asymmetrical patchy hyperintense lesions (arrows) in the periventricular and subcortical white matter of both cerebral hemispheres (1A to 1E) and the white matter of both cerebellar hemispheres (1F). FLAIR: fluid-attenuated inversion recovery; T2W: T2-weighted; MRI: magnetic resonance imaging

A 57-year-old male presented in April 2021 with a fever for one day, decreased consciousness, and repeated convulsions. At the emergency and casualty (E&C) department, he was unconscious. The Glasgow Coma Scale (GCS) score was eight (E2V1M5), without neck rigidity. He had received a second dose of the Sinopharm COVID-19 vaccine four weeks ago. He had no focal neurological deficits, pupillary abnormalities, or fundoscopic abnormalities. The plantar response was bilateral flexor. Our working diagnosis was meningoencephalitis. Empirical intravenous (IV) antibiotics, antivirals, corticosteroids (dexamethasone), and antiepileptics (levetiracetam) were started. The patient was nursed in a high-dependency unit (HDU). Relevant blood investigations were done; except for neutrophilic leukocytosis, other investigations reports were normal. The patient’s attendant did not give consent for the lumbar puncture and cerebrospinal fluid (CSF) study. The patient regained consciousness the next day. We did an MRI of the brain, which revealed multiple hyperintense lesions in different parts of the brain, suggesting ADEM (Figure 1).

Though the patient was already improving, we started standard treatment with ADEM: IV methylprednisolone (1 gram) daily for five days, followed by oral prednisolone in a tapering dose. Adequate hydration and nutrition were ensured. Electroencephalography (EEG) was normal. Except for antiepileptics, other medications were stopped. The patient recovered clinically completely and was discharged after 10 days. The patient was doing well during periodic follow-ups over the last year. He is asymptomatic now.

Case two

**Figure 2:**
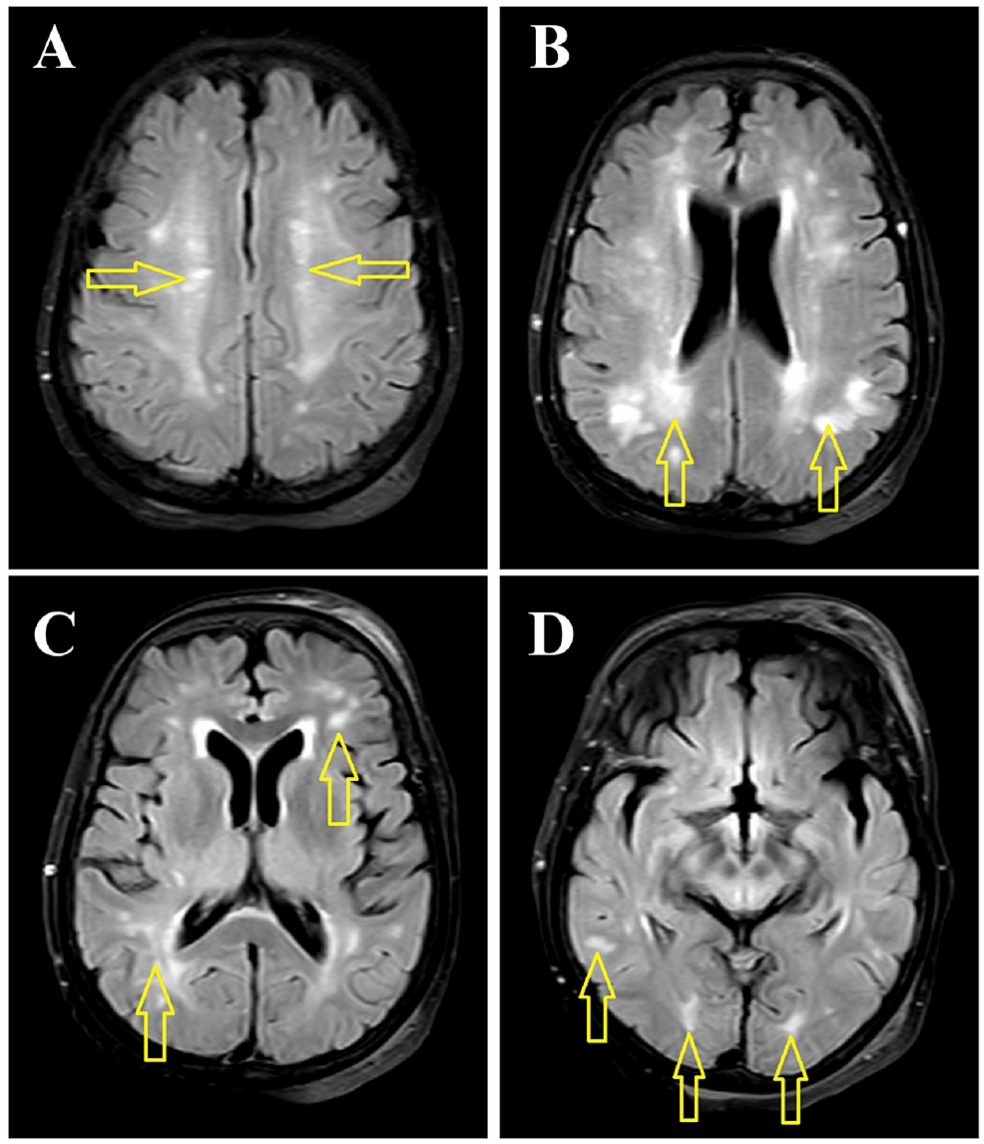
Axial FLAIR MR images (2A to 2D) of the brain at different levels show bilateral asymmetrical, discrete, and confluent hyperintense lesions (arrows) in the periventricular (1B, 1C) and subcortical white matter and centrum semiovale of both cerebral hemispheres (2A to 2D). FLAIR: fluid-attenuated inversion recovery; MR: magnetic resonance

In December 2021, we had our second case. A 60-year-old female developed a low-grade fever and repeated convulsions within six hours of receiving the second dose of the Sinovac COVID-19 vaccination. She had no adverse effects after the first dose of the COVID-19 vaccination. At the E&C, she was conscious but not oriented regarding time, place, or person. Other neurological examinations were normal. She was also treated empirically as a case of suspected meningoencephalitis initially. MRI of the brain was suggestive of ADEM (Figure 2).

IV methylprednisolone (1 g daily for five days) was started, and she recovered completely. Like in case one, this patient’s attendants also did not give consent for the CSF study. EEG revealed generalized slowing, suggestive of encephalopathy. She was diabetic, and except for raised blood sugar, her other relevant investigation findings were normal. She was discharged with oral antiepileptic medication. She came for follow-ups after one and three months of discharge. She was found asymptomatic during follow-up after three months of discharge.

Case three

**Figure 3:**
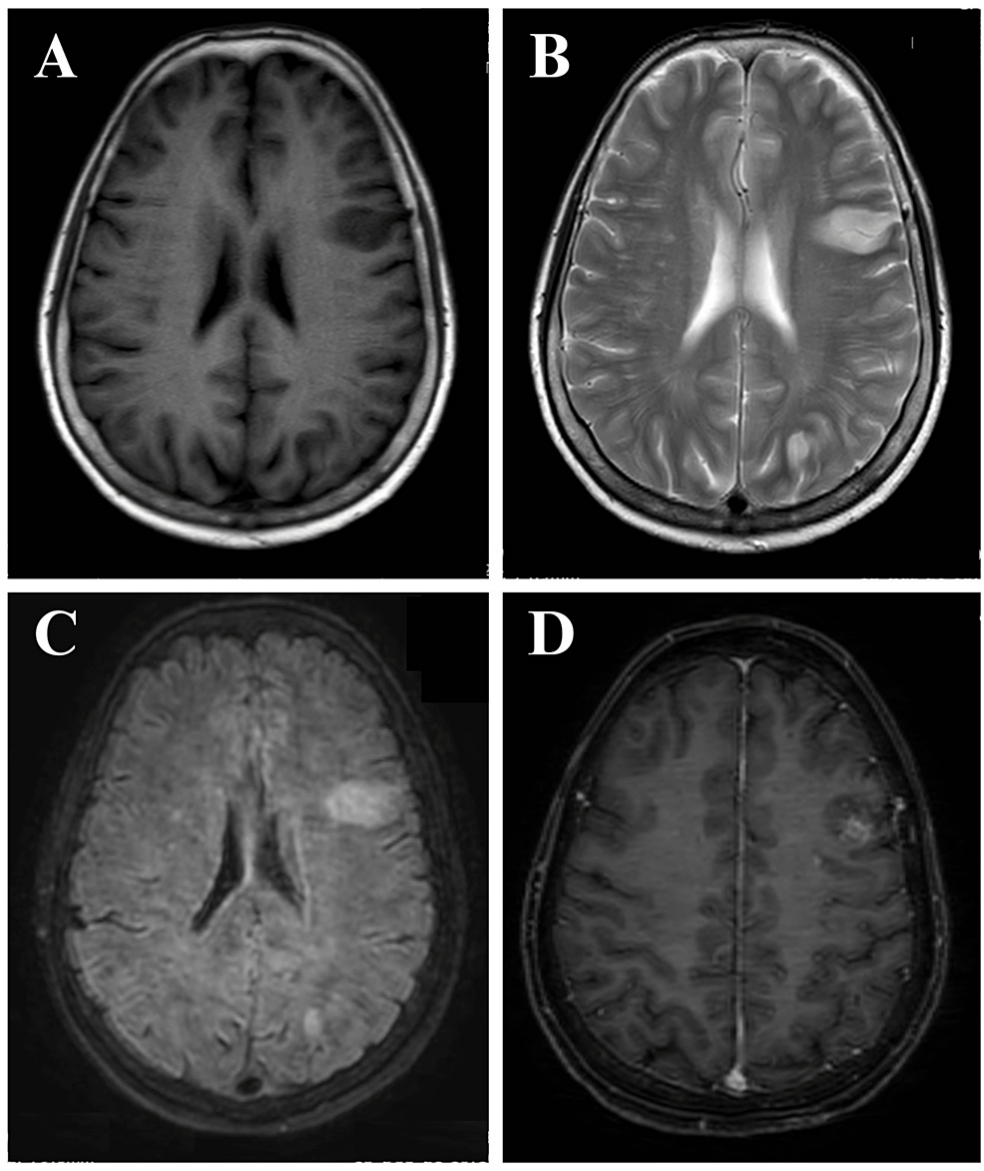
Axial T1W (3A), T2W (3B), FLAIR (3C), and post-gadolinium T1W (3D) MR images of the brain at different levels show multiple discrete T1 hypointense and T2 and FLAIR hyperintense lesions (arrows) of variable sizes in the periventricular and subcortical white matter of both cerebral hemispheres, with the largest lesion in the left frontal lobe involving the overlying cortex. A post-contrast scan shows nodular enhancement of the lesions (3D). T1W: T1-weighted; T2W: T2-weighted; FLAIR: fluid-attenuated inversion recovery; MR: magnetic resonance

A 69-year-old female was brought to the E&C with a fever and decreased consciousness for 10 days. Her symptoms started after two days of receiving the second dose of the Sinopharm COVID-19 vaccine. She developed a low-grade fever and an altered sensorium. She was initially treated empirically in a local hospital for a suspected case of meningoencephalitis. As her condition was not improving, she was referred to our center for further management. At the E&C, she was in a coma with a GCS of five (E1V1M3). Other neurological examination findings were unremarkable. The patient’s attendants signed the do-not-resuscitate (DNR) form, and the patient was treated conservatively in the HDU. The MRI of the brain was suggestive of ADEM (Figure 3).

One of the differentials was cerebral venous sinus thrombosis (CVST), as it may also occur after COVID-19 vaccination and present with similar clinical features. Our patient did not have any papilloedema. Features of raised intracranial pressure, including papilloedema, are usually present in CVST, especially when the patient is in a coma. The MRI of the brain showed no hemorrhage inside the lesions, which goes against the venous infarct. Venous infarcts usually spare the cortex, but lesions in our case involved the cortex. Considering all the above, we think it was more suggestive of ADEM. She was treated with IV methylprednisolone (1 g daily) for five days with no improvement. The patient developed aspiration pneumonia, and eventually, we lost the patient after two weeks.

Case four

A 53-year-old female presented to us with fever and myalgia within six hours of taking a booster dose of the Moderna COVID-19 vaccine. She previously completed her two doses of COVID-19 vaccination with the Sinopharm vaccine about eight months ago. On the third day of vaccination, she developed repeated convulsions. On admission, the patient was confused. She was treated with IV antibiotics, dexamethasone, and levetiracetam for a suspected case of meningoencephalitis. Her symptoms improved within 24 hours. There was mild neutrophilic leukocytosis with elevated C-reactive protein (CRP) of 52 mg/L (<10 mg/L). Serum IL-6 (interleukin-6) level was 28.6 pg/ml (up to 7 pg/ml). However, the serum ferritin level was within the normal range of 64.42 ng/ml (13-150 ng/ml). The MRI of the brain, electroencephalogram (EEG), and CSF study were also normal. Antibiotics were stopped after getting a normal CSF report. Dexamethasone was tapered gradually. She was discharged after seven days of complete recovery.

The brief descriptions of all cases are summarized in Table 1.

**Table 1:** Summary of clinical features, investigations, and treatment protocol of all cases. DM: diabetes mellitus; HTN: hypertension; ADEM: acute demyelinating encephalomyelitis; CSF: cerebrospinal fluid; mg/dl: milligrams per decileter; cmm: cubic millimeters

Traits	Case one	Case two	Case three	Case four
Age (years)	57	60	69	53
Sex	Male	Female	Female	Female
Time frame	Apr-21	Dec-21	Jan-22	Apr-22
Vaccination status	Received two doses of the Sinopharm vaccine at a four-week interval.	Received two doses of the Sinovac vaccine at a four-week interval.	Received two doses of the Sinopharm vaccine at a four-week interval.	The booster dose of the Moderna vaccine was previously completed after two doses of the Sinopharm vaccine six months ago.
Incubation period	Four weeks after the second dose	After about six hours of the second dose	Two days after the second dose	After six hours of the booster dose
Brief clinical history	Fever, repeated convulsions, and decreased consciousness	Fever and convulsions	Vertigo, vomiting, and decreased consciousness	Fever and myalgia followed by repeated convulsions
Other co-morbidities	None	DM	None	DM, HTN, and chronic stable angina
MRI brain findings	ADEM	ADEM	ADEM	Normal
CSF findings	Not done	Not done	Not done	Protein 42 mg/dl (15–45), sugar 88 mg/dl (50–80) Cell 02/cmm (0–5), lymphocytes, culture-negative
EEG	Normal (done after recovery)	Generalized slowing, suggesting encephalopathy	Not done	Normal (done after recovery)
Final diagnosis	ADEM	ADEM	ADEM	Acute reversible vaccine-related encephalopathy
Received treatment	Methylprednisolone	Methylprednisolone	Methylprednisolone	Dexamethasone
Prognosis	Complete recovery	Complete recovery	Death	Complete recovery

## Discussion

There are different types of COVID-19 vaccines available worldwide. In Bangladesh, a total of nine types of vaccines received approval [7]. There are numerous case reports already published regarding neurological complications of the COVID-19 vaccination. The highest percentage of complications was found with the AstraZeneca vaccine, followed by the Pfizer and Moderna vaccines [3]. Cerebral venous sinus thrombosis was the most common serious complication affecting the nervous system [3]. Encephalopathy and ADEM are also important complications reported in COVID-19-vaccinated individuals with significant morbidity and mortality [2,3,4].

We have found encephalitis (ADEM) or encephalopathy associated with three types of vaccines. These three vaccine types have separate mechanisms of action. The Moderna vaccine is an mRNA-based vaccine, whereas the Sinovac and Sinopharm vaccines are inactivated, low-virulent COVID-19 virus-based vaccines. The mRNA-based vaccines induce virus-specific proteins inside the human body, and our immune systems act against these proteins. The inactivated or attenuated virus-based vaccines directly trigger the defense system. As a result, both humoral and cell-mediated immunity are activated [2,8,9]. Similar to all other vaccines, these vaccines may also trigger inflammatory conditions like ADEM or acute encephalopathy.

Our patients developed symptoms within six hours to up to four weeks of vaccination. Among our four patients, one developed symptoms within a very short time after vaccination (case four). An elderly female developed a fever within six hours of receiving the Moderna vaccine and later developed convulsions. The patient responded well to antiepileptics and IV dexamethasone. The MRI of the brain and CSF studies were normal. Due to the acute presentation of the case, we treated her empirically for suspected acute meningoencephalitis with IV antibiotics, antivirals, and dexamethasone according to our hospital protocol. Later, the normal CSF findings and temporal relationship led to a diagnosis of possible vaccine-associated encephalopathy or acute reversible vaccine-related encephalopathy.

COVID-19 vaccines may be associated with hyperacute reversible encephalopathy related to cytokine storms [10,11]. Like the COVID-19 virus, COVID-19 vaccines trigger the immune response and may lead to a cytokine storm. This cytokine storm is associated with secondary organ dysfunction, including encephalopathy. The inflammatory markers are usually raised, including serum ferritin and IL-6 [12]. However, according to the U.S. Vaccine Adverse Event Reporting System (VAERS), in COVID-19 vaccine-related hyperacute reversible encephalopathy, cytokines, and inflammatory markers may not be raised in all cases [10]. Our case had raised CRP and IL-6. A CSF study usually does not reveal marked pleocytosis [12]. We have found normal CSF findings, which support our diagnosis. Corticosteroids and tocilizumab are the drugs of choice, especially in cytokine storms [12]. Our patient recovered rapidly, indicating a positive response to dexamethasone.

The other three cases (cases one, two, and three) had suggestive MRI findings of ADEM with variable incubation periods of six hours to four weeks after vaccination. The two patients with a short incubation period developed symptoms after the second dose of the vaccine. ADEM has been known to occur up to three months after the infection or vaccination. So, this immune encephalitis may also be due to their first dose. ADEM has similar presentations to infectious encephalitis. The patient may have a fever, confusion, loss of consciousness, seizures, cranial nerve deficits, a visual field defect, language disturbances, and features of meningism. Exclusion of other differentials like infectious autoimmune encephalitis and typical lesions in the MRI of the brain is usually diagnostic of ADEM [6,13]. An MRI of the brain usually reveals features of multifocal or diffuse white matter lesions. Lesions are usually bilateral, asymmetrical, large, and poorly demarcated. It may involve supratentorial and infratentorial regions of the brain or the spinal cord [6]. The patient should be followed up for at least three months from the symptomatic nadir for any relapse or recurrence. If the condition recurs or relapses, ADEM is unlikely [13]. CSF findings may reveal pleocytosis and raised protein. However, it may also be normal [6]. Myelin oligodendrocyte glycoprotein (MOG) antibodies are positive in a significant percentage of ADEM patients, especially in pediatric cases [6,14]. CSF oligoclonal bands and serum anti-aquaporin 4 antibodies are usually negative [6].

In all cases, COVID-19 PCR was negative. Connective tissue disease markers were negative. Metabolic derangements and infectious pathology were also excluded in all cases. We could not perform an autoimmune encephalitis panel, paraneoplastic autoantibodies, CSF oligoclonal antibodies, and other autoantibodies related to CNS demyelinating lesions, including myelin oligodendrocyte glycoprotein (MOG) antibodies and anti-aquaporin 4 antibodies, due to unavailability in our center. However, highly suggestive MRI brain findings and no recurrence or relapse in the three-month follow-up period strongly suggest ADEM is the likely diagnosis in our cases.

All the patients recovered completely with IV methylprednisolone for five days, except one patient. This patient developed aspiration pneumonia, and the next of kin (NOK) signed the DNR form. Unfortunately, the patient died. We also could not do the CSF study in two patients, as the patient and NOK did not give consent for lumbar puncture. We found abnormal EEGs in only one patient. One reason may be due to doing the EEG after several days when the patients were already improving.

After the confirmation of the diagnosis, ADEM cases are usually managed with high-dose IV steroids like methylprednisolone (1 g daily for three to five days), followed by oral prednisolone in a tapering dose. If the patient does not improve with pulse methylprednisolone, he can be treated with IVIg (intravenous immunoglobulin) or plasmapheresis. If the above modalities fail, IV cyclophosphamide can be tried [15].

A recent review article showed 54 cases of possible COVID-19 vaccine-related ADEM. Most of the cases were related to the first dose of the vaccine (more than 80%). The incubation period varied from 12 hours to 63 days. Most of the cases were related to the AstraZeneca vaccine. However, there were reported cases after almost all the available COVID-19 vaccine types [16]. Our cases will add more information related to COVID-19 vaccine-related ADEM.

As we have already mentioned, our cases have some limitations regarding investigations. However, the clinical profiles of the patients were strongly supportive of our diagnoses.

## Conclusions

The COVID-19 mass vaccination is an unprecedented event in the history of mankind. Every day, a handful of literary works are being published worldwide documenting the COVID-19 vaccine-related adverse effects. Among these complications, neurological manifestations are often deadly if not diagnosed and treated early. This case series will increase awareness among clinicians regarding ADEM and acute encephalopathy following COVID-19 vaccination.
